# Changes in Native Sentence Processing Related to Bilingualism: A Systematic Review and Meta-Analysis

**DOI:** 10.3389/fpsyg.2022.757023

**Published:** 2022-02-21

**Authors:** Patricia Román, Irene Gómez-Gómez

**Affiliations:** ^1^Departamento de Psicología, Universidad Loyola Andalucía, Seville, Spain; ^2^Loyola Behavioral Lab, Universidad Loyola Andalucía, Seville, Spain

**Keywords:** bilingualism, sentence processing, L2 to L1 influence, linguistic variation and change, systematic review, meta-analysis

## Abstract

The native language changes as a result of contact with a second language, and the pattern and degree of such change depend on a variety of factors like the bilingual experience or the linguistic level. Here, we present a systematic review and meta-analysis of works that explore variations in native sentence comprehension and production by comparing monolinguals and bilinguals. Fourteen studies in the meta-analysis provided information regarding the bilingual experience and differences at the morphosyntactic level using behavioral methods. Overall, we observed that first language processing is subject to small transformations in bilinguals that occur in sentence comprehension and production. The magnitude of the changes depended on bilingual experiences, but only length of residence in an L2 setting predicted the degree of change, where shorter length of residence was associated with larger changes. Results are discussed and related to the cognitive processes that potentially cause the transformations in the first language. The present work reveals some limitations in the field that should be addressed in future studies to better understand the mechanisms behind language attrition.

## Introduction

When learning a language, individuals often rely on their native language (L1) to facilitate the acquisition of the second one (L2), but learners also find that the characteristics of their L1 interfere when they are incongruent with those in the second language (Lardiere, 2009; Libben and Titone, 2009; Macizo et al., 2010; Paolieri et al., 2010; Casaponsa et al., 2015; Peristeri et al., 2018; Contemori et al., 2019, among others). Therefore, an extensive line of work in bilingualism has been devoted to exploring L1 influences on the L2.

Despite this interest, there is also evidence that contact with a second language transforms the processing of the L1 at different linguistic levels and carries a deviation from monolinguals (the so-called attrition, Schmid, 2010). For example, bilinguals are slower than monolinguals when they name pictures in their native language (e.g., Gollan et al., 2005), and they present more tip-of-the-tongue states than monolinguals (Stasenko and Gollan, 2019) what suggests that they have reduced access to words in their L1. In addition, there are conceptual shifts where bilinguals change how they connect words to meanings (Pavlenko, 2000, 2004; Ameel et al., 2009; Pavlenko and Malt, 2011). At the grammatical level, research has found that learning a second language may yield modifications too, including gender assignments (e.g., Kaushanskaya and Smith, 2016) and parsing preferences such as changes in the likelihood with which bilinguals attach a relative clause to a specific noun in ambiguous sentences (Dussias, 2003, 2004; Dussias and Sagarra, 2007). Finally, there are also differences in brain activity, even when behavioral performance is similar between monolinguals and bilinguals (e.g., Bice and Kroll, 2015; Román et al., 2015).

Several causes have been proposed to explain the differences between monolinguals and bilinguals in their native language. Some authors state that the reduction in accessibility to the L1 representations in bilinguals may be related to a lesser frequency of L1 use (e.g., weaker links hypothesis; Gollan et al., 2008). In fact, variables associated with a reduction in the L1 input such as L2 proficiency and immersion in a context where the L2 is spoken (Gollan et al., 2008; Monaghan et al., 2017) seem to moderate the changes observed in bilinguals across time in, for example, fluency and naming tasks (Linck et al., 2009; Baus et al., 2013).

Another explanation for the phenomenon is the transfer of L2 features (Costa and Sebastián-Gallés, 2015). Investigations show bidirectional transfer (L1 to L2 and L2 to L1) not only at the lexical but also at the grammar level, which, according to the data, seems to be less permeable to influences from the L2 (Andersen, 1982; Hicks and Domínguez, 2020). One example comes from bilinguals speaking a language that allows omission of pronouns and a language where pronouns are always present. In a study by Sorace (2000), Italian–English bilinguals increased their production of overt pronouns in Italian (a null-pronoun language), and they did so in locations that were more usual in their second language (English, an overt-pronoun language) than in their native one (for example, before the verb rather than post-verbal subject pronouns in Italian–English bilinguals).

Finally, research has extensively demonstrated that, in bilinguals, both languages are co-activated even in contexts where only one is necessary (e.g., Hatzidaki et al., 2011; Bobb et al., 2020). This co-activation involves the recruitment of capacity-limited cognitive resources and mechanisms of control aimed to avoid interference from the non-intended language that may result in differences in how bilinguals process their L1 (e.g., Titone et al., 2011; Misra et al., 2012). Several circumstances can increase a load of resources, for instance, the unbalance between languages proficiency (e.g., the unintended language being more dominant), the complexity of sentence structure, or the similarity across languages. In this regard, increased co-activation can cause facilitation (e.g., cognates, words that are similar in form and meaning) or interference (e.g., homographs, words that are similar in form differ in meaning). Also, individual differences in cognitive control and working memory (Cunnings, 2017a,b) might influence the magnitude of L1 variation^1^.

Regardless of the processes implicated in how the L2 influences the native language in bilinguals, L1 attrition is not a one-way road. Although, as mentioned above, the extent of L2 contact appears to be crucial in the occurrence and depth of changes in the L1, the L2 effects on the native language do not always seem constrained to high L2 proficiency, immersion, long-term exposure, or increased frequency of L2 use. Some studies reveal changes that emerge in adult learners after limited exposure to a new language in phonetic properties (Chang, 2012), lexicon (Baus et al., 2013; Bice and Kroll, 2015), and morphosyntax (Dussias et al., 2016), and with different time-courses and degrees of affection (Sorace, 2011). Again, the rapid variations observed in the L1 suggest that the native language is not a static entity, and in fact, such shifting is not necessarily linear or incremental. For example, Chang (2012) observed that native English speakers who were novice Korean learners presented after a few weeks of exposure to the L2 a phonological drift in their L1, that is, modifications that reflect the assimilation to phonetic characteristics of a different language. The author compared this group to experienced learners enrolled in the same course (Chang, 2013), and this latter group showed a reduced drift compared to their novice peers. Besides, re-immersion in the L1 environment may reverse the changes observed (Chamorro et al., 2015; Sorace, 2020), while, in other cases, the effects of the L2 contact may persist after individuals are no longer using it, and the duration of this influence diverges depending on the linguistic property under scrutiny (Linck et al., 2009).

The production and comprehension of sentences provide a rich and informative ground to explore the influence from L2 to L1. In a sentence, lexical, morphosyntactic, and pragmatic information interact, and individuals build upon these elements to convey or understand a message. Importantly, languages differ in terms of the weight that each of the aspects mentioned above has in a sentence and the information they provide, for example, case marking in German articles reveals the role of the subsequent noun in a sentence and is absent in Spanish or English. Concerning the influence of the L2 on L1 within sentence processing, linguistic levels differ in their degree of attrition after L2 acquisition, to give an example, bilinguals and monolinguals show similar responses to gender agreement violations in their native language but not to violations in verb combinations (Bergmann et al., 2015). Moreover, sentence production is more susceptible to cross-linguistic influences than comprehension (Runnqvist et al., 2013).

Although processes behind sentence production and comprehension partly coincide (e.g., Walenski et al., 2019), language production requires retrieval from memory, selection of intended representations, and speech planning processes subject to demands that may differ from reading or listening to sentences (Daneman and Green, 1986). Sentence production provides additional information too. Authors such as Schmid et al. (2013) consider that tasks where participants freely produce a discourse (for example, by asking them to report what they saw in an image or video fragment) allow bilinguals to display their entire repertoire without restrictions. All the above makes the study of sentences more informative than single-word studies about the circumstances that bilinguals face daily but more challenging to tackle.

Studied variables that modulate the size of L1 variation in sentence processing could be divided into those related to the bilingual experience *per se*, linguistic variables, and individual differences in cognition. Within the first category, language dominance (Sanoudaki and Thierry, 2015; Kasparian et al., 2017), proficiency (Opitz, 2010; Cherciov, 2011), and frequency of use (Schmid and Dusseldorp, 2010; Kasparian and Steinhauer, 2017) have been more extensively explored. The results have led researchers to considering explanations in terms of weakened representations in L1 because of a reduced input and greater co-activation and competition between linguistic representations (Steinhauer and Kasparian, 2020). In this sense, immersion in the L2 environment represents an extreme case of exposure to L2 and limited contact with L1. For example, Dussias and Sagarra (2007) compared Spanish monolinguals, Spanish–English bilinguals with limited immersion in their L2 context, and Spanish–English bilinguals with extensive immersion while reading sentences that included a relative clause and two antecedent nouns (as in “An armed robber shot the **sister** of the **actor** who was in the balcony”). In such cases, Spanish speakers have a preference to attach the relative clause (“who was in the balcony”) to the first noun (“the sister”), while native speakers of English prefer the second noun attachment (“the actor”). In their study, only bilinguals with long-term immersion in their L2 environment revealed an attachment in their L1 to the second noun, similar to native speakers of English.

Age of L2 acquisition (AoA) is another variable that has been associated with the degree of change in L1. AoA has been a matter of long debate in bilingual research under the assumption that while late bilinguals can master a second language, they hardly process morphosyntactical features the way monolinguals do (Wartenburger et al., 2003; Clahsen and Felser, 2006, but see Diependaele et al., 2011; Román et al., 2021). When considering its role in L2 to L1 influence, the question is whether some properties of the L1 become resistant to changes in late learners of L2 (Schmid and Köpke, 2017). Putting aside the case of heritage speakers (unbalanced bilinguals that learned their heritage language at home in a context where there is a dominant community language; see Benmamoun et al., 2013) that may not have fully consolidated their native language before they learn the second language, investigations addressing this matter are scarce (Karayayla and Schmid, 2019). Using semi-structured interviews, Karayayla and Schmid (2019) collected free speech samples from highly proficient Turkish–English bilinguals with an AoA that range from 7 to 34 y-o and compared the occurrence of complex syntactical forms to a group of Turkish monolinguals. They did not find a relation between the AoA and the structural complexity.

Another aspect of interest within the bilingual experience is the degree of overlap between languages. As mentioned above, the extent to which languages share properties can determine the co-activation of languages, facilitating (e.g., Domínguez, 2013) or interfering with the integration of both linguistic systems (Steinhauer and Kasparian, 2020). For example, Bernolet et al. (2007) investigated whether bilinguals were more likely to repeat a syntactical structure in a language (a passive) after they had been exposed to that same structure in another language (cross-linguistic priming) when a confederate described a picture. They had participants that spoke languages with different (English–Dutch) or similar word order (Dutch–German). While they observed priming in constructions where the word order matched, they did not when it differed. Nevertheless, research shows changes in bilinguals who speak languages with different parsing preferences (noun attachment in relative clauses; Dussias, 2003, 2004; Dussias and Sagarra, 2007).

Regarding individual differences in cognition, accounts based on limited capacities due to co-activation or susceptibility to interference (Cunnings, 2017a) predict that higher cognitive control and working memory will reduce processing difficulties in bilingual sentence parsing (but see Brothers et al., 2021). Following the same rationale, cues that facilitate successful retrieval of information will help to overcome interference and bias sentence processing, as observed in studies investigating relative clause attachment (Dussias and Sagarra, 2007) or interpretation of null/overt pronouns (Tsimpli et al., 2004) and, therefore, they might be preferred and used by bilinguals (Cunnings, 2017a,b).

So far, mixed and limited results blur the connection between the mechanisms proposed to cause attrition and the outcomes in the studies, in part due to the heterogeneity of the bilingual experience. Here, we try to put together research on sentence processing to shed some light and pave the path to future inquiries.

### The Present Study

Research demonstrates that the linguistic system is not a static unit but rather a malleable and adaptive organization that integrates novel entries at different levels (de Bot et al., 2007; Kroll et al., 2014; Kaan et al., 2019) and the context of sentence processing provides a broad but complex ground to investigate how various linguistic representations interact in the bilingual mind. In the present study, we present a systematic review and meta-analysis study to explore the current evidence on how the bilingual experience changes the processing of sentences in the native language to unravel different patterns and the factors that underlie them.

## Methods

We performed a systematic review and meta-analysis following the Preferred Reporting Items for Systematic Reviews and Meta-Analysis (PRISMA) guidelines (Moher et al., 2009). The protocol of this study was previously registered at PROSPERO on May 16*^th^*, 2021 (registration number CRD42021245042).

### Search Strategies

The search was conducted in Web of Science (WOS), PsycINFO, PubMed, and Scopus from database inception to March 3, 2021, and the strategy comprised keywords and text words related to bilingualism, attrition, and sentence processing and comprehension. We first piloted the search strategy in WOS and then adapted it to run across PsycINFO, PubMed, and Scopus (see Supplementary Material). We also screened reference lists of included studies and previous reviews on the topic. There were no language or year restrictions.

### Eligibility Criteria

Following PICO criteria (population, intervention, comparator, and outcome), our inclusion criteria were as follows: the population was bilinguals, here broadly defined as participants tested in their native tongue but proficient in a second language; individuals suffering from any linguistic deficit, children, and heritage speakers were excluded; regarding intervention or exposures, we included cross-linguistic influence from the second language (L2) to the first language (L1) in morphosyntactic processing, and the comparator was morphosyntactic native language processing in bilingual participants contrasted to monolinguals’ (participants with minimal to no experience with a language different from their native tongue) in both sentence comprehension and sentence production assessed by behavioral measures. Finally, the outcome of this study was the influence of the L2 on the L1, as seen through different psycholinguistic tasks and behavioral measures.

### Selection of Studies

Two independent reviewers screened the title and abstract (LG and IGG) and full text to assess the eligibility of the studies. A third reviewer made the final decision (PR) in case of disagreements. The software system used for recording decisions was Rayyan (Ouzzani et al., 2015).

### Data Extraction

Again, two independent reviewers (PR and IGG) extracted the data and resolved discrepancies by consensus. We collected information related to the first author, publication year, total sample size, bilingual sample size, monolingual sample size, target population (pair of languages and language experience), cognitive processes studied (comprehension and production), task, and measures. More specifically, behavioral measures such as reaction times, accuracy, acceptability ratings in comprehension and pauses, errors, diversity, and complexity in production were included. Seven articles were discarded because of unavailable data, and information from groups of heritage speakers or children was not included in the analyses.

### Statistical Analysis

Quantitative data were analyzed using the Comprehensive Meta-Analysis (CMA) software package, V.3 (CMA) (Borenstein et al., 2013) and Stata release 14.2. (StataCorp, 2015). First, standardized mean differences (SMDs) were calculated from each group’s mean and SD, and when unavailable, we calculated SMDs from sample size and *p*-value. Then, CMA was used to obtain the equivalent SMDs. Finally, the pooled SMDs for all studies and their 95% CIs were estimated. Some studies only reported *p*-values corresponding to interactions or group effects that included heritage speakers groups and were discarded from the analyses.

The SMD between bilingual and monolingual participants was used as effect size using Hedges’ *g* formula (Hedges, 1981) for small sample sizes. A negative value indicated higher scores in morphosyntactic tasks for the monolingual group in sentence comprehension and sentence production. Following previous evidence, the sign of the SMD was inverted for reaction time, errors, and pause measures so that the SMD went in the same direction as other measures such as accuracy in which the higher the mean, the better the performance (e.g., Beaussart et al., 2018). Interpretation of the resulting SMD followed Cohen’s proposal: 0.20 as a small effect size, 0.50 as a medium effect size and, 0.80 as a large effect size (Cohen, 1988). In addition, random-effects models were used for pooling effect sizes assuming studies included heterogeneous populations that may differ from each other.

Inspection of heterogeneity was carried out through visual inspection of the forest plot, Q Cochran’s statistic, and *p*-value. Additionally, heterogeneity was quantified by the *I*^2^ index and its 95% CI, and interpretation of the *I*^2^ index was subject to the following level and percentages: unimportant heterogeneity (0–40%), moderate heterogeneity (30–60%), substantial heterogeneity (50–90%), and considerable heterogeneity (75–100%) (Higgins and Green, 2011; Borenstein et al., 2017).

We employed the Duval and Tweedie trim-and-fill procedure (Duval and Tweedie, 2000), the Begg and Mazumdar rank correlation (Begg and Mazumdar, 1994), and the Egger test (Stuck et al., 1998) to explore publication bias.

Furthermore, sensitivity analyses were conducted to explore SMD when Cohen’s *d* and the fixed-effects model were used. Finally, possible SMD variations were examined independently for studies focused on sentence comprehension, sentence production and for those exploring syntactic and morphological processing.

Finally, subgroup analyses were performed using a mixed-effects model for the categorical moderators AoA (early or late bilinguals), immersion context (either L1 or L2), length of residence (LoR) (short, long or no immersion), task modality (visual, auditorily and audiovisual), and structure congruence (similar and dissimilar across languages).

There was more than one effect size for all studies, and, therefore, the data might be considered dependent. Because of such dependence, we performed a meta-regression analysis on the continuous data with robust variance estimates (RVE; Hedges et al., 2010; Tanner-Smith and Tipton, 2014) using the *Robumeta* command in Stata. When fewer than four degrees of freedom were present, the results were considered unreliable (Tanner-Smith et al., 2016). Following previous research, covariates included in the RVE meta-regression were LoR, AoA, and L2 proficiency (e.g., Schmid and Dusseldorp, 2010). We performed both bivariate meta-regression analysis (including only one of the three independent variables in each meta-regression analysis) and multivariate meta-regression analysis (including all covariates in the same meta-regression analysis).

## Results

### Study Selection

**FIGURE 1:**
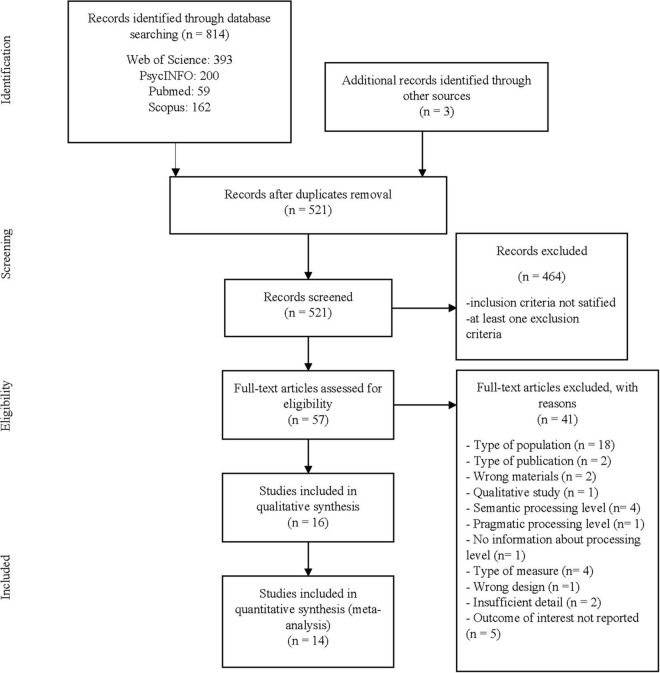
PRISMA flow diagram of the studies.

A total of 817 articles were identified through searching databases and other sources. After eliminating duplicates, 521 published studies and unpublished doctoral dissertations remained, and we screened the title and abstract. Fifty-seven studies initially met the inclusion criteria and were subject to a full-text inspection. This procedure yielded 16 articles that were included in the systematic review (see Figure 1 for details about the exclusion criteria). We finally included 14 studies with 14 independent group comparisons and 81 effect sizes for the meta-analysis calculations. Two studies (Castro et al., 2017; Dragoy et al., 2019) were excluded from the meta-analysis because they did not provide the needed statistics to calculate SMD.

### Study Characteristics

A total of 1,044 participants were included across 16 studies in the systematic review (see Table 1 for study characteristics). Of them, 486 were bilinguals, and 412 were monolinguals. Ten studies included late bilinguals (studies IDs: 2, 5, 8, 9, 10, 11, 12, 14, 15, and 16), three studies included only early bilinguals (studies 3, 7, and 13), another two studies included both late, and early bilinguals (studies 1 and 6) and one study did not provide information regarding the age of acquisition (study 4). Regarding the pair of languages under scrutiny, two studies investigated Spanish–Swedish bilinguals (studies 2 and 3). The following pairs were investigated in one study each: Turkish–German (study 1), English–Spanish (study 12), Spanish–English (study 10), Italian–English (study 16), German–English (study 14), Brazilian Portuguese–European Portuguese (study 4), Chinese–Korean (study 5), Russian–German (study 6), Turkish–English (study 7), Greek–Swedish (study 8), Russian–Hebrew (study 9), German–Dutch (study 11), German–Italian (study 15), German–Spanish (study 15), Greek–English (study 16), and Spanish–Catalan (study 13). Ten studies measured sentence comprehension (studies 1, 2, 3, 4, 6, 7, 8, 9, 10, and 11) while five studies explored sentence production (studies 5, 12, 14 and 15). One study collected data from both sentence production and comprehension (study 16). All studies used behavioral measures.

**TABLE 1:** Characteristics of the studies included in the systematic review.

					Target population				
								
Study ID	References	Total sample size	Bilingual sample size	Monolingual sample size	Language experience	Pair of languages	Cognitive process	Task	Measures
1	Arslan et al., 2015	61	LB: 20; EB: 19	22	Early and late Turkish–German bilinguals and Turkish monolinguals	Turkish–German	Sentence comprehension	Grammatical evidentiality	Behavioral (ACC and RT)
2	Bylund and Ramírez-Galan, 2014	59	39	20	Late Spanish–Swedish bilinguals and Spanish monolinguals	Spanish–Swedish	Sentence comprehension	Grammaticality Judgment test	Behavioral (AR)
3	Bylund et al., 2010	40	25	15	Early Spanish–Swedish bilinguals and Spanish monolinguals	Spanish–Swedish	Sentence comprehension	Grammaticality Judgment test	Behavioral (AR)
4	Castro et al., 2017	98	32	34	Brazilian Portuguese–European Portuguese bilinguals, Brazilian Portuguese monolinguals	Brazilian Portuguese–European Portuguese	Sentence comprehension	Acceptability judgment task	Behavioral
5	Chunpeng and Hee-Don, 2017	40	20	20	Late Chinese–Korean bilinguals and Chinese monolinguals	Chinese–Korean	Sentence production	Composition task	Behavioral (errors)
6	Dragoy et al., 2019	60	30	30	Early and late Russian–German bilinguals and Russian monolinguals	Russian–German	Sentence comprehension	Grammaticality Judgment test	Behavioral (ACC)
7	Gürel, 2015	54	27	27	Early Turkish–English bilinguals and Turkish monolinguals	Turkish–English	Sentence comprehension	Acceptability judgment task	Behavioral (AR)
8	Kaltsa et al., 2015	91	25	18	Late Greek–Swedish bilinguals and Greek monolinguals	Greek–Swedish	Sentence comprehension	Self-paced listening sentence-picture matching task	Behavioral (RT and ACC)
9	Laufer and Baladzhaeva, 2015	81	44	21	Late Russian–Hebrew bilinguals and Russian monolinguals	Russian–Hebrew	Sentence comprehension	Grammaticality judgment task	Behavioral (AR)
10	Liceras and Senn, 2009	44	12	20	Late Spanish–English bilinguals and Spanish monolinguals	Spanish–English	Sentence comprehension	Acceptability judgment task; Clitic-triggered attachment	Behavioral (AR)
11	Ribbert and Kuiken, 2010	90	52	38	Late German–Dutch bilinguals and German monolinguals	German–Dutch	Sentence comprehension	Grammaticality judgment task	Behavioral (errors)
12	Ribes and Llanes, 2015	35	15	20	Late English–Spanish bilinguals and English monolinguals	English–Spanish	Sentence and lexical production	Storytelling test; C-Cloze test	Behavioral (pauses and FO)
13	Román et al., 2015	33	16	17	Early Spanish–Catalan bilinguals and Spanish monolinguals	Spanish–Catalan	Sentence comprehension	Acceptability judgment	Behavioral (ACC)
14	Schmid, 2014	126	53	53	Late German–English bilinguals, English learners of German and German monolinguals	German–English	Sentence production	Spontaneous speech sampling	Behavioral (ACC)
15	Schmitz et al., 2016	53	I/G: 10; S/G: 8	I: 10; S: 7	Late Italian–German bilinguals, late Spanish–German bilinguals, Spanish monolinguals, and Italian monolinguals	Italian–German; Spanish–German	Sentence production	Spontaneous speech sampling	Behavioral (FO)
16	Tsimpli et al., 2004	79	I/E: 20; G/E: 19	I: 20; G: 20	Late Italian–English bilinguals, late Greek–English bilinguals, Italian monolinguals, and Greek monolinguals	Italian–English/Greek–English	Sentence production and comprehension	Headlines task; picture verification task	Behavioral (FO)

*Groups within studies that do not meet the criteria are not included in the table.*

*Bilingual sample size: E/R, English and Russian speakers; R/E, Russian and English speaker; LB, late bilingual; EB, early bilinguals; I/G, Italian and German speakers; S/G, Spanish and German speakers; I/E, Italian and English speakers; G/E, Greek and English speakers.*

*MonolingualMonolingual sample size: E, English speakers; R, Russian speakers; I, Italian speakers; S, Spanish speakers; G, Greek speakers.*

*Measures: RT, reaction times; ACC, accuracy; AR, acceptability ratings; FO, frequency of occurrence.*

### Differences Between Bilinguals and Monolinguals in L1

The meta-analysis was calculated based on 81 effect sizes reported in 14 articles. The pooled SMD was −0.155 (95% CI, −0.301 to −0.009; *p* < 0.001). There was substantial heterogeneity across studies (*I*^2^ = 79.1%; 95% CI, 74% to 83%), and it reached significance (*Q*_80_ = 382.99; *p* < 0.001). These results showed a statistically significant difference between monolinguals and bilinguals in L1 morphosyntactic processing, and according to Cohen’s proposal, the effect size was small.

### Publication Bias

**FIGURE 2:**
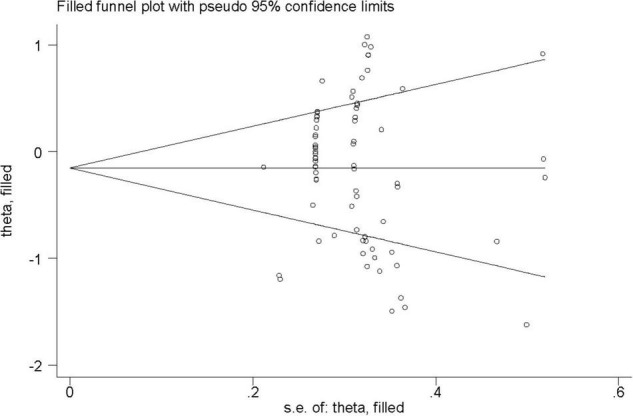
Funnel plot.

Results of the Egger’s test (bias, −0.907; 95% CI, −4.082 to 2.268; *p* = 0.571) and Begg and Mazumdar’s test (*z* = 1.02; *p* < 0.314) indicated no publication bias. The Duval and Tweedie procedure did not impute any missing studies, and the SMD did not change [SMD, −0.155; (95% CI, −0.301 to −0.009); *p* < 0.038]. As a result, there is no evidence of publication bias. The funnel plot is shown in Figure 2.

### Sensitivity Analysis

The pooled SMD based on the 81 effect sizes revealed little change when Cohen’s *d* [SMD, −0.157 (95% CI, −0.305 to −0.008; *p* = 0.039)] and the fixed-effects model [SMD, −0.144 (95% CI, −0.210 to −0.078; *p* = 0.000)] were used. The effect size remains small in both cases, and the differences between monolingual and bilingual speakers remain statistically significant. If studies on morphological processing were independently subject to analyses, the SMD increased considerably, and the differences between monolingual and bilingual speakers remain statistically significant [SMD, −0.882 (95% CI, −1.167 to −0.598; *p* < 0.001)]. However, when studies on syntactic processing [SMD, −0.011 (95% CI, −0.155 to 0.133; *p* = 0.879)], comprehension tasks [SMD, −0.165 (95% CI, −0.339 to −0.008; *p* = 0.061)], and production tasks [SMD, −0.124 (95% CI, −0.397 to 0.148; *p* = 0.372)] were analyzed separately, the SMD varied with no significant differences between monolingual and bilingual groups. Table 2 summarizes the results of the sensitivity analysis.

**TABLE 2:** Sensitivity analysis.

Analysis	No. of effect sizes *(k)*	SMD	95% CI	*p*	*I*^2^ (95% CI)
Effectiveness	81	−0.155	−0.301 to −0.009	0.038	79.1% (74%–83%)
Cohen’s *d* test	81	−0.157	−0.305 to −0.008	0.039	79.02% (74%–83%)
Fixed effect model	81	−0.144	−0.210 to −0.078	< 0.001	79.1% (74%–83%)
Including comprehension task studies only	59	−0.165	−0.339 to 0.008	0.061	81.1% (76%–85%)
Including production task studies only	22	−0.124	−0.397 to 0.148	0.372	72.1% (57%–82%)
Including syntactic processing studies only	68	−0.011	−0.155 to 0.133	0.879	73.8% (67%–79%)
Including morphological processing studies only	13	−0.882	−1.167 to −0.598	< 0.001	67.8% (43%–82%)

### Subgroup Analysis

Table 3 depicts the results for the subgroup analysis. The studies that did not report information about some of the variables of interest (AoA, immersion context, immersion duration, modality of presentation, structure similarity) comprised a no-information group included in the corresponding subgroup analysis to explore their role in the outcome.

**TABLE 3:** Subgroup analysis.

Subgroup analysis	No. of effect sizes *(k)*	SMD	95% CI	*p* ^†^	*I* ^2^	Between-group heterogeneity^‡^
**Type of task**						
Comprehension	59	–0.165	−0.339 to 0.008	0.061	81.1%	*Q*_1_ = 0.46, *p* = 0.500
Production	22	–0.124	−0.397 to 0.148	0.372	72.1%	
**AoA (type of bilingualism)**						
Early bilingualism	25	0.022	−0.103 to 0.147	0.734	26.2%	*Q*_2_ = 18.05, *p* < 0.001
Late bilingualism	45	–0.193	−0.424 to 0.038	0.102	83.5%	
No information	11	–0.423	−0.932 to 0.086	0.104	84.9%	
**Context**						
Immersed in L1	1	0.206	−0.462 to 0.874	0.545	–	*Q*_1_ = 1.06, *p* = 0.302
Immersed in L2	80	–0.159	−0.307 to −0.012	0.035	79.3%	
**LoR**						
Short	2	–1.148	−1.798 to −0.498	0.001	48.6%	*Q*_1_ = 17.76, *p* < 0.001
Long	79	–0.130	−0.276 to 0.016	0.080	78.5%	
**Modality**						
Auditory	23	–0.202	−0.569 to 0.166	0.282	85.4%	*Q*_2_ = 5.32, *p* = 0.070
Visual	42	–0.189	−0.372 to −0.006	0.043	77.7%	
Audiovisual	16	0.005	−0.262 to 0.272	0.970	65.5%	
**Structure congruence**						
Different	43	–0.134	−0.294 to 0.027	0.103	67.8%	*Q*_4_ = 109.21, *p* < 0.001
Similar	25	0.190	−0.053 to 0.434	0.125	75.8%	
Not applicable	5	–1.054	−1.405 to −0.703	0.001	21.0%	
Collapsed	1	–1.163	−1.611 to −0.715	0.001	–	
No information	7	–0.751	−1.348 to −0.154	0.014	84.7%	

*^†^Significance tests in which for each subgroup, the null hypothesis is that SMD = 0.*

*^‡^Q-values represent the comparison of subgroup means based on a chi-square distribution in which the null hypothesis is that the effect size is the same for all subgroups.*

We inspected subgroups associated with characteristics of bilingual experience. There were statistically significant differences between subgroups investigating early, late bilinguals, and a no-information group (*Q*_2_ = 18.05, *p* < 0.001) with greater effect sizes observed in studies without information related to AoA (*k* = 11), while studies including late AoA (*k* = 45) presented a greater effect compared to those comprised of bilinguals with early acquisition of the L2 (*k* = 25). However, none of the individual effects in each group was significant (all *p*’s > 0.05). When considering immersion, no differences appeared between articles exploring bilinguals immersed in their L1 (*k* = 1) and those exploring bilinguals immersed in their L2 (*k* = 80), but this last group of studies presented a significant effect size (*p* = 0.035). Finally, when looking at LoR in the L2 environment, we found statistically significant differences between short and long LoR studies (*Q*_1_ = 17.76, *p* < 0.001), and the effect was significant in the short LoR (*k* = 2), but it did not reach significance in the long LoR subgroup (*k* = 79; *p* = 0.080).

Exploration of the similarity of the structures between languages revealed that effect sizes significantly differed when we compared studies using similar and dissimilar features between languages (similar, dissimilar, collapsed, n/a, and no-information, *Q*_4_ = 109.21, *p* < 0.001), but differences between monolinguals and bilinguals were not significant in the group of studies that used either similar or dissimilar structures. As seen in Table 3, effect sizes were larger if the languages’ characteristics were collapsed (*k* = 1).

Investigating the modality of stimuli presentation, research dealing with auditory, visual, and audiovisual material did not significantly differ between modalities, and the effect size was statistically significant only in the group with visual presentation (*k* = 42; *p* = 0.043).

### Meta-Regression

Meta-regression analyses with RVE (Hedges et al., 2010; Tanner-Smith and Tipton, 2014) were performed. We considered the effect of each continuous moderator individually (LoR, L2 proficiency, or L2-AoA; bivariate meta-regression) over the SMD, and the results indicated a significant difference in the effect size magnitude associated with LoR (β = 0.040 [95% CI, 0.013 to 0.066]; *p* < 0.016). However, according to Tanner-Smith et al. (2016), the *p*-value was untrustworthy because the degrees of freedom were less than four. No significant differences were observed for proficiency in L2 and L2-AoA variables (see Table 4).

**TABLE 4:** Coefficient statistics of meta-regression analysis with RVE estimates on the association between SMD and other covariates.

	Beta	95% CI	*p*
**Bivariate meta-regression**			
LoR	0.040	0.013 to 0.066	0.016
Proficiency in L2	0.050	−0.474 to 0.574	0.730
AoA	–0.001	−0.050 to 0.050	0.964
**Multivariate meta-regression**			
LoR	–0.031	−0.046 to 0.108	0.254
AoA	0.019	−0.064 to 0.102	0.499

*LoR, length of residence; AoA, age of acquisition.*

When the effect of all covariates (LoR and L2-AoA) were considered simultaneously (multivariate meta-regression) in the same RVE meta-regression model, LoR and age of acquisition did not predict the effect size magnitude. L2 proficiency had to be excluded from the analysis due to missing values.

## Discussion

The work presented here addresses the changes that the native language undergoes as a consequence of contact with a second language in bilinguals. Our aim was twofold: we wanted to explore circumstances that lead to variations in the L1 and find a connection between the explanatory accounts of bilingual/monolingual differences in native processing and the data. To do so, we employed a systematic review and meta-analysis of research in sentence comprehension and production comparing monolingual and bilingual performance. Next, we summarize our results, and then we will try to link them with cognitive processes that might be behind changes in the native processing of sentences.

The systematic review comprised 16 studies, and 14 were used in the meta-analysis. Overall, results showed that individuals who speak more than one language were subject to variations in their native tongue with a small and significant effect size. As we strove to tell apart L1 changes connected to the main facets of bilingual sentence processing, subgroups analyses were used. No differences were observed between comprehension and production, and importantly, if analyses considered each group of studies separately, monolinguals and bilinguals did not display different behavior. Regarding the bilingual experience, differences were statistically significant in studies that compared monolinguals to bilinguals immersed in their L2 and bilinguals with a short LoR in a context where the second language is spoken. In the meta-analysis regression, the continuous variables L2 proficiency and AoA did not predict the effect size observed in sentence processing; following the subgroups analysis, shorter LoR predicted wider differences between monolinguals and bilinguals in their L1. All in all, these findings suggest that L1 processing may be subject to small, qualitatively different variations across the bilingual experience rather than accumulative (Schmid and Karayayla, 2020; Steinhauer and Kasparian, 2020), and all the factors should be taken into consideration when addressing the patterns of bilingual processing in their native language.

### Comprehension and Production Studies

Sentence production is less explored than comprehension despite the stress that recent approaches give to considering both production and comprehension and its interdependence to feed language models (Pickering and Garrod, 2013; Dell and Chang, 2014). Our work reflects this trend in research, with a higher number of studies targeting comprehension.

Within comprehension, most of the reviewed studies used acceptability/grammaticality judgments (12 studies; 10 included in the meta-analysis, see Table 1). In an investigation by Schmid and Dusseldorp (2010), German monolinguals outperformed German–English bilinguals and German–Dutch bilinguals in verbal fluency, the C-test, and a film re-telling task; however, their behavior was similar in an auditorily grammatical judgment task. Although their results may reveal stable L1 knowledge at this linguistic skill in bilinguals, the authors do not discard the hypothesis that the sentences could be easy for the readers. Also, the use of brain markers as participants read sentences may reveal differences that the offline acceptability judgments do not, even in low complexity sentences (see Román et al., 2015). For example, Italian–English bilinguals and Italian monolinguals in Kasparian et al. (2017) had to rate the grammaticality of eight-word sentences in Italian that could include local or non-local agreement violations while neurophysiological activity was recorded (for example, “Il lavatore torna dalla fabbrica sporco di grasso,” *The* ***workers***_*plural*_ *returns*_*singular*_ *from the factory dirty*_*singular*_ *with grease*). They did not find a group effect or group interaction at the acceptability ratings, but groups differed in reaction times and the neurophysiological patterns. Together with neurophysiological methodologies, other online measures like eye-tracking or self-paced reading may be more sensitive than offline acceptability judgments to catch differences between groups (e.g., self-paced listening, Kaltsa et al., 2015, in our review; for a deeper discussion on online measures in the field, see Marinis, 2003; Roberts, 2012). The prevalence of the acceptability-ratings task and behavioral measures within our sample of studies might be behind the lack of a subgroup effect.

Research dealing with sentence production, on the other hand, employed spontaneous speech/writing sampling (speech, Schmid, 2014; Schmitz et al., 2016; writing, Chunpeng and Hee-Don, 2017), storytelling (Ribes and Llanes, 2015), and a headlines task, which provides a verb, a noun phrase and an adverbial expression that participants have to use to produce a sentence describing a picture (Tsimpli et al., 2004). When present, differences in these studies showed that bilinguals tended to be slower and employed syntactical structures that were permitted in their L1 but preferred in their L2, suggesting that variation, rather than attrition or loss is a term better suited for L1 changes in individuals that speak more than one language (Schmid, 2014; Schmitz et al., 2016). Although a separate analysis of bilinguals vs. monolinguals in sentence production studies did not show a significant effect size, the production pattern reveals co-activation and transfer of L2 features in the bilingual grammar, as we will explain later.

### Bilingual Experience

The magnitude of effect sizes differed between groups within AoA, and LoR, as seen in the subgroups analysis, but were only statistically significant when looking at studies that included immersion in L2 and short LoR. Nevertheless, LoR was the only continuous variable predicting differences between monolinguals and bilinguals in the bivariate meta-regression. In Schmid and Dusseldorp’s (2010) study mentioned above, one of their objectives was investigating variables that predicted L1 attrition. Only LoR predicted attrition in free speech (in lexical diversity and errors) using a film re-telling task. Some articles in the present review that did not investigate the relation of attrition with LoR used a similar procedure. For example, Schmid (2014) had a subgroup of 20 late German/English bilinguals with a minimum LoR in Canada of 9 years but up to four decades (mean and standard deviation not provided) that did not differ from their monolingual peers in morphosyntactic variables. In our meta-regression, lower LoR is associated with larger differences between bilinguals and monolinguals and grants the need for further research, including an earlier (and shorter) range of LoR in sentence production.

More recently, Schmid and Karayayla (2020) explored the role of LoR in comprehension. They collected sentence production data from 92 Turkish-English bilinguals (collapsed including heritage speakers and therefore excluded from our review and meta-analysis) about their L1 maintenance and acquisition within a wide range of age at onset of bilingualism (AaO; from birth to adulthood). The data indicated that LoR predicted morphosyntactic complexity in L1, but in a direction opposite to our results, the longer the residence in a context where the second language is used, the lower the proficiency in L1. Importantly, they observed that the effects were more evident in early than late bilinguals. Because in the present work 11 of the 14 studies included late bilinguals, it is necessary to assume that variations in the L1 are qualitatively different between bilingual experiences and likely the result of different cognitive processes.

In spite of that, the continuous variables LoR, L2 proficiency, and AoA did not predict variation in the L1 in the multivariate meta-regression. One potential explanation is that bilinguals can use cues of different nature to compensate for differences, masking the effect of the variables of interest at the behavioral level examined here. For example, in a study collecting neurophysiological data, Kasparian and Steinhauer (2017) used an acceptability judgment task in Italian with relative clause structures that could be temporarily ambiguous (garden-path as in “Il poliziotto che i ladri arresta registra i nomi,” *The policeman that the thieves* ***arrests*** *registers the names*). The sentences were grammatical in Italian, but some were ungrammatical in English (as in the example) and less preferred garden-path structures in Italian. As expected, bilinguals found the grammatical sentences in Italian but ungrammatical in English as less acceptable than monolinguals. Moreover, the authors predicted a P600 in both groups (greater in bilinguals) to the verb in the relative clause (*arresta*), commonly found in garden-path sentences as an index of syntactic difficulty, and an N400 related to difficulties in semantic integration because they introduced strong semantic cues (policeman-thieve-arrest) that did not conflict with the structure. Italian monolinguals, which rely more on semantic information, evinced an N400, while the bilinguals did not show this component but a greater P600, as anticipated if they used, like English monolinguals, the strict word order preferably than semantic cues.

One problem in our study that may prevent us from finding a stronger impact of L2 proficiency is using the bilingual term and the collection of L2 proficiency data broadly to cover as many studies as possible. Some authors have warned about the implications that the way we conceive, and measure bilingualism have on the diversity of outcomes we obtain in our growing field of knowledge (Green and Abutalebi, 2013; Luk and Bialystok, 2013; Ooi et al., 2018; Anderson et al., 2020; Kremin and Byers-Heinlein, 2021). In the pool of articles reviewed here, proficiency tests go from subjective reports, including those with questions about how much effort it takes to use a language (Liceras and Senn, 2009), to objective measures and placement tests (for example, TOEFL for English proficiency, Chang, 2009; Gürel, 2015; telc for Turkish, Arslan et al., 2015). In addition, some works did not contain proficiency information (e.g., Kaltsa et al., 2015), or provided ranges (e.g., Bylund and Ramírez-Galan, 2014). Under such circumstances, there was enough data to consider proficiency as a categorical variable but impeded the continuous data to be used as recommended for the meta-regression (Bialystok, 2018; De Cat et al., 2018; Gunnerud et al., 2020).

### Connection to Explanatory Accounts

As mentioned in the introduction, researchers have considered three leading causes behind the patterns observed in bilingual L1 processing: language co-activation in bilinguals, cross-linguistic transfer, and a reduced frequency of L1 use (Costa and Sebastián-Gallés, 2015; Cunnings, 2017a; Schmid and Köpke, 2017).

Cunnings (2017a) proposes that retrieval interference/facilitation lies behind differences in bilingual comprehension. Retrieval interference appears because working memory is a capacity-limited entity (Baddeley, 2013), and co-activation of languages increases the demands when bilinguals have to select one representation from those retrieved from long-term memory and integrate it with the incoming information. In agreement with this idea, Chunpeng and Hee-Don (2017) tested Chinese/Korean bilinguals reporting the use of both languages daily, and therefore more prone to co-activation. Their sample presented difficulties retrieving words in written composition and spent a longer time completing the task compared to Chinese monolinguals. Apart from that, in their research, grammar differences were mainly related to transfer from their L2 (Korean word order, punctuation, among others), similar to English/Spanish bilinguals in Ribes and Llanes (2015) that produced more subordinate constructions allowed in English but preferred in Spanish, and more pauses. Bilinguals may then become “opportunistic” speakers under co-activation contexts selecting representations that alleviate their cognitive load and languages cooperate rather than interfere (Beatty-Martínez et al., 2020).

A prediction derived from the above is that similarities across languages may influence bilingual processing, increasing co-activation, interfering or facilitating processing, or making bilinguals opting for the common structures in sentence processing. However, we have not observed differences between groups with similar and dissimilar structures, and none of the analyses that independently tested similarity and dissimilarity in bilingual vs. monolingual performance was significant. In this respect, co-activation might happen not only when the use of specific structures sharing properties across languages spread activation to nodes in the L2, but also when using the L1 in broader contexts such as L2 immersion (as seen confirmed in the meta-analysis) or when co-activation is locally induced (e.g., watching a movie in the L2; Elston-Güttler et al., 2005). This indicates that any context that prompts co-activation may increase the chances of altered processing even in the absence of shared properties.

In association with this idea, Green and Abutalebi (2013) proposed the adaptive control hypothesis. According to this approach, bilinguals find themselves in different contexts, and each posits specific demands to which the reader/speaker adapts. That is, bilinguals’ L1 (and L2) is not only subject to differences in the long run but varies depending on the demands of the environment (e.g., the accent of the interlocutor, unilingual workplace, bilingual community, discourse complexity, etc.). Whenever co-activation creates competition between language schemas, there will be a need for processes that handle interference and select the desired linguistic representations (Green and Abutalebi, 2013). Such processes, on the one hand, use limited resources that may be unavailable for efficient processing (Perfetti and Stafura, 2014; Li and Clariana, 2019) and, on the other hand, make competing representations less accessible through inhibition (e.g., Levy et al., 2007). These processes engaged in controlling interference might explain the direction of LoR effects in our data; at short LoR, the native language is dominant in late bilinguals and L2 usage is expected to trigger inhibitory processes to overcome the interference and facilitate retrieval of the weaker L2 representations. When bilinguals try to retrieve their L1 later, it takes time to access the suppressed representations in the L1. As asymmetry between languages decreases, inhibition is no longer needed (Levy et al., 2007).

Also, predictions from this approach are that reading and producing sentences will show either an impairment (for example, an increasing number of pauses, reduced processing speed) or compensation (for example, using simpler structures, using a limited number of cues, repetition of more accessible structures). As discussed earlier, bilinguals in some of the studies reviewed here present more pauses and longer time to produce written or verbally sentences in their L1 (Ribes and Llanes, 2015; Chunpeng and Hee-Don, 2017). Additionally, it is expected that bilinguals (and monolinguals) will have to overcome co-activation and inhibition, and that succeeding in doing so must be more difficult in constructions that demand resources allocation, such as complex grammatical forms (e.g., evidentiality marking in Turkish, Arslan et al., 2015) or pronominal referential relations in sentence comprehension (Li and Clariana, 2019). In fact, pronominal resolutions have been studied in the context of language attrition, considering that difficulties in this type of structure may arise due to the need to coordinate different interfaces (grammatical and discourse information; Sorace, 2011; Chamorro et al., 2015). Giving support to this view, we have found research in which bilinguals do not have a strong preference toward a pronominal assignment in the presence of more than one antecedent noun as monolinguals have (Kaltsa et al., 2015; Castro et al., 2017; but see Liceras and Senn, 2009; Schmitz et al., 2016) and show preferences that place less cognitive demands on the reader (low-attachment in L1 in both English–Spanish and Spanish–English bilinguals, Dussias, 2003, 2004), thereby supporting the notion of bilinguals as strategists that adapt to the requirements of their linguistic and cognitive context.

Another factor that may alter L1 processing is the cross-linguistic transfer (Costa and Sebastián-Gallés, 2015). In this regard, we have seen that bilinguals show in their L1 a bias to structures and interpretations that appear frequently in their L2 (examples in production are Ribes and Llanes, 2015; Chunpeng and Hee-Don, 2017; in comprehension, Dussias and Sagarra, 2007). Changes in the L1 sentence processing can appear as a lack of preference for both L1 and L2 biases too, similar to what Kaltsa et al. (2015) and Castro et al. (2017) observed. Specifically, in two experiments, Kaltsa et al. (2015) employed a self-paced listening task with a sentence-picture matching to test pronoun resolution in Greek. They presented sentences such as “I γiaγia xeretise tin kopela otan afti pernuse to δromo” (*The old lady greeted the girl when she crossed the street*) where the antecedent of the overt pronoun in the subordinate clause “afti” (*she*) could be the subject (old lady) or the direct object (the girl). In Greek, the null subject is the default, and the subject antecedent is preferred under such conditions, while a non-subject antecedent (the girl in the example) is preferred in overt subject pronoun as the one in the example. In this research, Greek–Swedish bilinguals with long LoR were expected to choose more subject antecedents in overt pronoun conditions than monolinguals, but their performance should be similar in null pronoun conditions (Swedish is a non-null subject language). Monolinguals showed differences between null and overt pronoun conditions, but bilinguals did not. Despite this outcome, Kaltsa and colleagues consider that cross-linguistic transfer is not causing their results because a group of older monolinguals presented a performance closer to bilinguals.

Finally, a frequency-based account highlights a reduced L1 input that weakens and biases activation toward L2 properties (Gollan et al., 2005). One way to explore this effect would have been to include relative frequency of use across languages in our analyses, but only a few had information about it (Liceras and Senn, 2009; Castro et al., 2017), and they did not explore its impact. Immersion length may switch the frequency balance between L1 and L2, and studies exploring parsing preferences that differ across languages are helpful since language biases may progressively shift with exposure. However, the results in our meta-regression with shorter LoR showing larger effects say otherwise. In two studies, Spanish–English bilinguals with long L2 immersion (Dussias and Sagarra, 2007) and Spanish–English bilinguals with shorter L2 immersion (Dussias, 2004) but similar proficiency change from L1 to L2 preferences while highly proficient Spanish–English bilinguals immersed in their L1 maintained the monolingual routines (Dussias and Sagarra, 2007). Such results could suggest that frequency-related modifications occur rapidly and appear in readers with shorter exposure to L2 but also that there is more than one mechanism underlying variations across bilingual groups in agreement with evidence that shows a non-linear L1 variation (Schmid and Karayayla, 2020). Other processes such as the need for L1 inhibition in an L2 setting cannot be ruled out, and ERPs studies point to a more significant role of competition in grammar differences instead of a reduced frequency in L1, at least in the early stages of immersion in an L2 context (for a review, see Steinhauer and Kasparian, 2020).

Although these explanations do not exclude each other and may act simultaneously, they do predict different outcomes under different conditions. Therefore, more research is necessary to separate their respective effects and the relative weight they have on bilinguals’ L1 at several stages and situations.

## Conclusion

To sum up, learning an L2 involves mutual influence between languages that vary qualitatively and quantitatively across time and experience, most likely in response to differences in demands of the environment and the cognitive processes recruited to deal with them.

In the present work, we targeted morphosyntactic processing. Some researchers consider that attrition occurs mainly at the lexical level in comprehension and production and that when grammatical rules are concerned, it affects aspects related to lexical retrieval (Schmid and Fägersten, 2010). Nonetheless, we have observed that bilingual deviations from monolinguals are evident though small in morphosyntax, even in individuals immersed in their second language but without knowledge in their L2 (Laufer and Baladzhaeva, 2015). It is important to note that studies targeting morphosyntax often imply the interplay of distinct linguistic subskills, as seen above in Kasparian and Steinhauer (2017), where semantic constraints influence bilingual parsing of garden-path sentences. Experimental designs that dissociate effects across linguistic levels will help to clarify differences in the processes subserving L2 influences in the L1.

Other variables not addressed here may influence the cognitive processes involved in sentence processing, resulting in differences between monolinguals and bilinguals in their native language. The two reasons behind this absence are the lack of consistency in gathering such data among studies and selecting contrasts between monolinguals and bilinguals instead of different groups within bilinguals. Further research will need to attend to these variables. For example, none of the experiments reviewed collected data about individual differences in either cognitive control (conflict detection, inhibition, cognitive flexibility, or task switching) or working memory, which impeded directly testing the association of cognitive resources in dealing with language co-activation. Besides, here the target contrast was comparing bilinguals to monolinguals. Hopp and Schmid (2013) warned of the difficulties that using monolinguals as a reference group entails, given that the mere existence of two languages in the bilingual mind provides a qualitatively different ground. In this sense, it seems more appropriate to compare early and late bilinguals who reach similar proficiency or investigate individual differences while matching bilingual experience. While we acknowledge the validity of Hopp and Schmid’s statement, we consider that there is a value in our work to address the distinct mechanisms involved in dealing with two languages and their relation with several aspects of the bilingual experience.

Apart from the above, the results presented require cautious interpretation. Although data dependence was controlled in the meta-regression analysis with RVE (Hedges et al., 2010; Tanner-Smith and Tipton, 2014), subgroups analyses, sensitivity, and main effect analyses did not control it. Besides that, we did not assess the risk of bias of the included studies. Aside from the methodological limitations, reported/collected information regarding the bilingual experience was quite heterogeneous across studies what prevented us from having a more extensive and detailed exploration of factors impacting language processing.

Despite these limitations, the present work followed the PRISMA guidelines; thus, the search of the studies was carried out in the most relevant databases using a wide variety of terms without restrictions regarding the year of publication or language, giving the systematic review and meta-analysis a high sensitivity. Additionally, two independent researchers went through the entire screening process, selection, and extraction of the characteristics. Last but not least, there was no evidence of publication bias, and we analyzed sensitivity, which contributes to the robustness of the results found.

Finally, some recommendations can be derived from our work. Methodologically speaking, offline measures, when used, should be accompanied by online measures that provide more sensitivity and information regarding the cognitive processes involved. In addition, because sentence processing implies the interaction of several types of information, the inclusion of conditions that allow isolating the influence of independent variables is encouraged. Regarding the target population, the study of cross-linguistic influence requires (1) a clear definition of terms like “proficiency”; (2) detailed data collection of measures and experience with both languages to have a profile that considers the balance between L1 and L2, and (3) going beyond monolinguals as a group of reference and consider bilingual groups that differ in several dimensions to explore the variables that affect changes in the L1.

## Data Availability Statement

The original contributions presented in the study are included in the article/Supplementary Material, further inquiries can be directed to the corresponding author.

## Author Contributions

PR conceived the study. PR and IG-G prepared the strategy search, conducted the systematic literature search, screened studies for eligibility together with Laura Giraldo and Raquel Román, extracted data, contributed to the manuscript preparation, and approved the final version. IG-G conducted the statistical analyses. Both authors contributed to the article and approved the submitted version.

## Conflict of Interest

The authors declare that the research was conducted in the absence of any commercial or financial relationships that could be construed as a potential conflict of interest.

## Publisher’s Note

All claims expressed in this article are solely those of the authors and do not necessarily represent those of their affiliated organizations, or those of the publisher, the editors and the reviewers. Any product that may be evaluated in this article, or claim that may be made by its manufacturer, is not guaranteed or endorsed by the publisher.

## Footnotes

1 Here the term L1 variation refers to deviations from the normative use of the L1 in monolinguals.
