# Supplementary material for: Changes in Native Sentence Processing Related to Bilingualism: A Systematic Review and Meta-Analysis
Source: Front Psychol. 2022 Feb 21;13:757023. doi: 10.3389/fpsyg.2022.757023 (PMC8898929; doi:10.3389/fpsyg.2022.757023)
Supplement: Supplementary file 1 [file Data_Sheet_1.docx]

**Supplementary materials**

**Search strategies**

Database: Web of Science (WOS); Search date: 15/03/2021

AB=(bilingual* OR non-native OR L2) AND AB=(monolingual* OR native OR L1) AND AB=(attrition OR crosslinguistic* OR transfer OR cross-influenc*) AND AB=(“sentence processing” OR “sentence comprehension” OR “sentence production” OR gramma* OR semantic*)

Database: PsychINFO; Search date: 15/03/2021

AB (bilingual* OR non-native OR L2) AND AB (monolingual* OR native OR L1) AND AB (attrition OR crosslinguistic* OR transfer OR cross-influenc*) AND AB (“sentence processing” OR “sentence comprehension” OR “sentence production” OR gramma* OR semantic*)

Database: PubMed; Search date: 05/04/2021

("bilingual*"[Title/Abstract] OR "non-native"[Title/Abstract] OR "L2"[Title/Abstract]) AND ("monolingual*"[Title/Abstract] OR "native"[Title/Abstract] OR "L1"[Title/Abstract]) AND ("attrition"[Title/Abstract] OR "crosslinguistic*"[Title/Abstract] OR "transfer"[Title/Abstract] OR "cross influenc*"[Title/Abstract]) AND ("sentence processing"[Title/Abstract] OR "sentence comprehension"[Title/Abstract] OR "sentence production" [Title/Abstract] OR "gramma*"[Title/Abstract] OR "semantic*"[Title/Abstract])

Database: Scopus; Search date: 15/04/2021

( ( ( TITLE ( bilingual* ) ) OR ( ABS ( bilingual* ) ) ) OR ( ( TITLE ( non-native ) ) OR ( ABS ( non-native ) ) ) OR ( ( ABS ( l2 ) ) OR ( TITLE ( l2 ) ) ) ) AND ( ( ( TITLE ( monolingual ) ) OR ( ABS ( monolingual ) ) ) OR ( ( TITLE ( native ) ) OR ( ABS ( native ) ) ) OR ( ( TITLE ( l1 ) ) OR ( ABS ( l1 ) ) ) ) AND ( ( ( TITLE ( attrition ) ) OR ( ABS ( attrition ) ) ) OR ( ( TITLE ( crosslinguistic* ) ) OR ( ABS ( crosslinguistic* ) ) ) OR ( ( ABS ( crosslinguistic* ) ) OR ( TITLE ( transfer ) ) ) OR ( ( TITLE ( cross-influenc* ) ) OR ( ABS ( cross-influenc* ) ) ) ) AND ( ( ( TITLE ( "sentence processing" ) ) OR ( ABS ( "sentence processing" ) ) ) OR ( ( TITLE ( "sentence comprehension" ) ) OR OR ( ( TITLE ( "sentence production" ) ) ( ABS ( "sentence comprehension" ) ) ) OR ( ( TITLE ( gramma* ) ) OR ( ABS ( gramma* ) ) ) OR ( ( TITLE ( semantic* ) ) OR ( ABS ( semantic* ) ) ) )

**Table S1**

*Inclusion and exclusion criteria*

| **Criteria** | **Inclusion criteria** | **Exclusion criteria** |
| --- | --- | --- |
| Population | Bilinguals | Individuals suffering from any linguistic deficit, children or heritage speakers |
| Intervention/ exposure | Crosslinguistic influence from L2 to L1 | Other types of crosslinguistic influences |
| Comparator | Monolingual performance in sentence comprehension and production tasks | Other comparators |
| Outcome | Influence of the L2 on the L1 | Other outcomes |
| Type of measure | Behavioral | Other types of measures |
| Type of processing level | Syntactic & morphological processing levels | Other types of processing level |
| Language | All | None |
| Publication year | All | None |

**Table S2**

*Outcomes or measures according to the type of processing level and number of effect sizes*

| **Processing level** | **Outcome/Measures** | **Nº effect sizes** |
| --- | --- | --- |
| Syntactic | Reaction time | 6 |
|  | Accuracy | 13 |
|  | Acceptability ratings | 25 |
|  | Matching | 4 |
|  | Listening times | 5 |
|  | Pauses per minute | 1 |
|  | Frequency of occurrence | 13 |
|  |  |  |
| Morphological | Reaction time | 2 |
|  | Accuracy | 3 |
|  | Errors | 4 |
|  | Acceptability ratings | 4 |
